# Harnessing the phyllosphere microbiota of wild foxtail millet for designing beneficial cross-kingdom synthetic communities

**DOI:** 10.1093/ismeco/ycaf066

**Published:** 2025-05-03

**Authors:** Xiaoyu Zai, Feng Zhu, Meicheng Zhao, Xianmin Diao, Fusuo Zhang, Francisco Dini-Andreote, Chrats Melkonian, Marnix H Medema, Jos M Raaijmakers, Viviane Cordovez, Chunxu Song

**Affiliations:** State Key Laboratory of Nutrient Use and Management, College of Resources and Environmental Sciences, China Agricultural University, 100193 Beijing, China; National Academy of Agriculture Green Development, China Agricultural University, 100193 Beijing, China; Key Laboratory of Plant-Soil Interactions, Ministry of Education, China Agricultural University, 100193 Beijing, China; National Observation and Research Station of Agriculture Green Development, 057250 Quzhou, Hebei, China; Key Laboratory of Agricultural Water Resources, Hebei Laboratory of Agricultural Water-Saving, Center for Agricultural Resources Research, Institute of Genetics and Developmental Biology, The Innovative Academy of Seed Design, Chinese Academy of Sciences, 050021 Shijiazhuang, China; Key Laboratory of Agricultural Water Resources, Hebei Laboratory of Agricultural Water-Saving, Center for Agricultural Resources Research, Institute of Genetics and Developmental Biology, The Innovative Academy of Seed Design, Chinese Academy of Sciences, 050021 Shijiazhuang, China; Institute of Crop Sciences, Chinese Academy of Agricultural Sciences, 100081 Beijing, China; Institute of Crop Sciences, Chinese Academy of Agricultural Sciences, 100081 Beijing, China; State Key Laboratory of Nutrient Use and Management, College of Resources and Environmental Sciences, China Agricultural University, 100193 Beijing, China; National Academy of Agriculture Green Development, China Agricultural University, 100193 Beijing, China; Key Laboratory of Plant-Soil Interactions, Ministry of Education, China Agricultural University, 100193 Beijing, China; National Observation and Research Station of Agriculture Green Development, 057250 Quzhou, Hebei, China; Department of Plant Science & Huck Institutes of the Life Sciences, The Pennsylvania State University, University Park, PA 16802, United States; The One Health Microbiome Center, Huck Institutes of the Life Sciences, The Pennsylvania State University, University Park, PA 16802, United States; Bioinformatics Group, Wageningen University & Research, 6708 PB Wageningen, The Netherlands; Theoretical Biology and Bioinformatics, Science for Life, Utrecht University, 3584 CS Utrecht, The Netherlands; Bioinformatics Group, Wageningen University & Research, 6708 PB Wageningen, The Netherlands; Department of Microbial Ecology, Netherlands Institute of Ecology, 6708 PB Wageningen, The Netherlands; Institute of Biology, Leiden University, 2333 BE Leiden, The Netherlands; Department of Microbial Ecology, Netherlands Institute of Ecology, 6708 PB Wageningen, The Netherlands; State Key Laboratory of Nutrient Use and Management, College of Resources and Environmental Sciences, China Agricultural University, 100193 Beijing, China; National Academy of Agriculture Green Development, China Agricultural University, 100193 Beijing, China; Key Laboratory of Plant-Soil Interactions, Ministry of Education, China Agricultural University, 100193 Beijing, China; National Observation and Research Station of Agriculture Green Development, 057250 Quzhou, Hebei, China

**Keywords:** domestication, plant microbiome, green foxtail, foxtail millet, biotic stress

## Abstract

Understanding the interplay between mechanisms in plant microbiome assembly and functioning of wild ancestors has led to the proposal of a novel strategy to enhance resilience to the (a)biotic stresses of domesticated crops. The challenge is determining how to harness the diverse microbiota of wild crop ancestors in their natural habitats in order to design effective synthetic microbial communities (SynComs) that reconstitute specific microbiome-associated plant phenotypes. In this study, we profiled the phyllosphere microbiota of wild green foxtail collected from seven geographically diverse natural ecosystems and showed that variations in soil parameters and climatic conditions as well as plant genetic distance significantly correlated with bacterial and fungal community compositions. Environmental selection and dispersal limitation differently governed the assembly of bacterial and fungal communities with distinct habitat niche breadth. Specific bacterial and yeast genera were identified as core phyllosphere taxa of wild green foxtail millet on the basis of their abundance and prevalence across the seven sampling sites. Moreover, several genera of bacteria (*Bacillus*, *Pantoea*, *Methylobacterium*) and yeast (*Vishniacozyma*, *Filobasidium*, *Sporobolomyces*) displayed significant correlations with the abundances of one or more foliar pathogenic fungi, in particular fungi of the genus *Alternaria*. Subsequent isolation and characterization of these bacterial and yeast genera allowed the design of cross-kingdom SynComs that protected domesticated foxtail millet from leaf infections by *Alternaria alternata*. These results provide fundamental insight into the mechanisms governing the phyllosphere microbiota assembly of a wild crop ancestor across large geographic scales and a practical framework to leverage this fundamental knowledge for the design of SynComs that mitigate the biotic stress of the domesticated crop.

## Introduction

Plant-associated microbiomes of crop wild progenitors have been recently proposed as a largely unexplored resource for enhancing plant tolerance to climate change conditions in modern agriculture [1, 2]. Green foxtail (*Setaria viridis*) is the progenitor of foxtail millet (*Setaria italica*) and displays great tolerance to (a)biotic stress conditions [3, 4]. Domesticated foxtail millet is also tolerant of multiple environmental stresses, including soil salinity and moderate drought. The value of domesticated foxtail millet as a “functional crop” relative to other cereal crops (e.g. wheat, rice, and maize) is also due to its diverse nutritional attributes, with contents such as high protein, essential amino acids, unsaturated lipids, dietary fiber, vitamins, minerals, and antioxidants, [5–8].

The phyllosphere (i.e. the aerial parts of plants, including leaves, flowers, stems, fruits, and pollen [9, 10]) constitutes one of the largest surface habitats supporting microbial life [11]. Phyllosphere microorganisms can exert beneficial functions for plant growth and health [12–14]. The most important factors determining the composition and temporal dynamics of phyllosphere microbiota include geographic locations (e.g. soil factors, climate, topography) and plant traits (e.g. plant species, genotypes within a species, leaf area, nutrients) [15–19]. In the context of plant genotypic effects and in particular domestication, several studies have compared the composition of phyllosphere microbiomes between crop species and their wild ancestors. For example, leaf microbial communities of wild rice differed taxonomically across locations and from the leaf microbial communities of cultivated rice, with bacterial genera such as *Methylobacterium*, *Sphingomonas*, *Phaeosphaeria*, and *Khuskia* more prevalent in wild than in cultivated rice [20]. These and other findings suggest that wild crop relatives harbor compositionally different phyllosphere microbiomes across geographical scales as well as a subset of microbial taxa that are found consistently in the phyllosphere of wild plant species and their domesticated counterparts [20–22]. Over the past decade, the concept and relevance of “core microbiomes” has gained significant traction pertaining to compositional stability relative to the resident microbiome [23]. The ability of core microbiota to colonize and survive in the phyllosphere of genetically diverse germplasm of a given plant species has been recognized as an important trait for the design of beneficial synthetic microbial communities (SynComs) for sustainable crop production [15–17, 20]. To date, however, little is known regarding the mechanisms governing the assembly of the phyllosphere microbiota of wild crop relatives in natural habitats and how increased knowledge in this area can be leveraged to reveal methods for enhancing tolerance of the domesticated crops to (a)biotic stresses.

In this study, we sampled leaves of wild green foxtail (*S. viridis*) populations at seven sites distributed across the center of origin of this plant in China [24] and investigated the impact of environmental factors (i.e. soil physicochemical properties and climate factors) as well as host factors (i.e. plant genetic diversity based on single-nucleotide polymorphisms) on the assembly of bacterial and fungal communities in the phyllosphere. We hypothesized that the phyllosphere of wild green foxtail harbors core microbial taxa that exert beneficial effects on the stress resilience of domesticated foxtail millet. In brief, our results revealed significant correlations between the abundances of core bacterial and yeast taxa with putative foliar fungal pathogens in wild green foxtail plants. Going from correlation to causation, we isolated and characterized core bacterial and yeast taxa to design cross-kingdom SynComs and tested their efficacy in suppressing leaf infections of domesticated foxtail millet caused by the fungal pathogen *Alternaria alternata*. By collecting fundamental information on the phyllosphere microbiome assembly of the wild progenitor, we successfully reconstructed a pathogen-suppressive phyllosphere microbial consortium for domesticated foxtail millet. This study provides a framework to explore and exploit the functional potential of microbiota of wild crop relatives as a strategy to mitigate biotic stresses in modern-day agricultural crops.

## Materials and methods

### Sampling of green foxtail leaves

**Figure 1:**
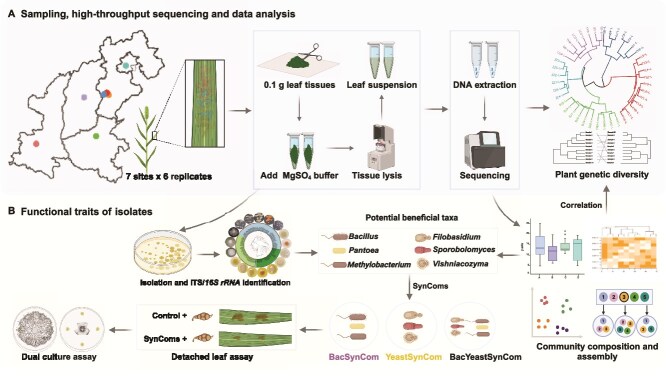
Flow charts of sampling, data analysis, identification and functional verification of potential beneficial microbial taxa. **A**. Plant genetic diversity and phyllosphere microbiome of green foxtail was analyzed by collecting leaf samples from seven sites in China. **B**. Isolation, taxonomic and antagonistic characterization of phyllosphere bacterial and yeast isolates.

Green foxtail (*Setaria viridis*) plants exhibiting high genetic diversity and specific accessions are distributed in northern China, where the Shanxi, Shaanxi, Henan, and Hebei provinces are the main sources of green foxtail germplasm [24]. We selected seven sites for collection of leaf samples of green foxtail growing wild in their native habitats [24]. Within each site, six patches, spaced 5–10 km apart, were chosen as replicates. Ten neighboring green foxtail plants at the heading stage within each patch were selected and pooled into a single sample (replicate). In total, there were 42 leaf samples (7 sites × 6 replicates) and 42 bulk soil samples. Samples were stored at −80°C and used for DNA extraction (Fig. 1A).

### Soil physicochemical analysis

Following standard analysis methods described by Bao [25], bulk soil samples were collected for soil physicochemical analyses, including pH, soil organic carbon (SOC), total phosphorus (TP), total nitrogen (TN), total potassium (TK), total carbon (TC), nitrate nitrogen (${\text{NO}_{3}}^{-}$−N), ammonium nitrogen (${\text{NH}_{4}}^{+}$−N), available phosphorus (AP), and available potassium (AK) (Supplementary Table S1). Climate data, including mean annual temperature (MAT), accumulated temperature (AT), relative humidity, mean annual precipitation (MAP), accumulated sunshine duration (AS), and annual cumulative solar radiation (AR) were sourced from the China Meteorological Data Network (http://data.cma.cn/) and are detailed in Supplementary Table S1.

### DNA extraction of leaf samples

To avoid soil contamination, leaf samples were gently washed with sterile deionized water, and any excess of water was removed by blotting the leaves on sterile filter paper. Although this process is a standard procedure for processing phyllosphere samples, it may lead to the depletion of some microbial taxa from the leaf samples. Nevertheless, we applied this methodology consistently for all leaf samples to ensure reliable comparisons in cross-sample analyses. Subsequently, 100 mg of fresh leaf tissue was weighed in a 2-ml tube with two sterile steel balls (diameter = 4 mm). The tube was immersed in liquid nitrogen for 1 minute and then ground for 2 minutes at a rate of 1600 for 2 cycles by use of a SPEX Geno/Grinder 2010 for tissue lysis. Leaf DNA was extracted according to the instructions of the DNeasy® PowerSoil® Pro Kit (QIAGEN, Germany; part number 47016). The quality of extracted DNA was determined by 1% agarose gel electrophoresis, and the DNA concentration and purity were measured using a NanoDrop 1000 spectrophotometer (Thermo Scientific). Leaf DNA samples were stored at −80°C for subsequent sequencing of *5S rRNA*, *knotted1* (*kn1*), and *16S rRNA* genes, and an internal transcribed spacer region (ITS).

### Sanger sequencing of *5S rRNA* and *knotted1* plant genes

To confirm species identification as green foxtail, specific primers targeting the housekeeping genes *5S rRNA* (forward primer: 5′-GGACCTCCTGCGAAG TCCT-3′ and reverse primer: 5′-CCCATCCGTGTACTACTCTC-3′) and *kn1* (kn211-402F: 5′-TCAGAACTTTTGGCCGTGGGT-3′ and kn612-402R: 5′-GAGATGGACAGCGAGTTGAGC-3′) were amplified and sequenced via Sanger sequencing [26]. Polymerase chain reaction (PCR) amplification was performed in a 20-μl system containing 2 μl 10 × Ex Taq Buffer, 1.6 μl 2.5 mM deoxynucleotide triphosphate, 1 μl of each primer (5 μm), 0.2 μl 5-unit Ex Taq Polymerase, 100 ng template DNA, and 3.7 μl double-distilled H_2_O. The PCR conditions were as follows: initial denaturation at 95°C for 5 min, followed by 25 cycles of denaturation at 95°C for 30 s, annealing at 56°C for 30 s, extension at 72°C for 1.5 min, and a final extension step at 72°C for 10 min. PCR products were purified from a 1.5% (w/v) agarose gel after electrophoresis. Further purification and quantification were performed using an AxyPrep DNA Gel Extraction Kit (Axygen Biosciences, Union City, USA) and a QuantiFluor™-ST Fluorometer (Promega, Fitchburg, USA) following the manufacturers’ instructions. Purified amplicons were subjected to Sanger sequencing at Majorbio (Shanghai, China). Phylogenic analysis of *5S rRNA* and *kn1* gene sequences was performed using the command “qiime phylogeny align-to-tree-mafft-fasttree” in QIIME 2 (Quantitative Insights Into Microbial Ecology 2, version 2021.2) and viewed in iTOL [27]. Sixty *5S rRNA* and *kn1* housekeeping gene sequences were used in the phylogenetic analysis, including 42 samples obtained and sequenced in this study, 14 samples from different *Setaria* genera as documented by Zhao *et al*. (2013) [26], 3 established *S. viridis* cultivars (Q23, Q19, Q4) from He *et al*. (2023) [3], and an outgroup, *Zea Mays* (GenBank: DQ351339).

### Whole-genome sequencing of green foxtail

The sequencing library was prepared using the NEB Next® Ultra™ DNA Library Prep Kit (New England Biolabs, Ipswich, MA, USA) following the manufacturer’s guidelines, with index codes added to each sample. Genomic DNA was sonicated to a size of 350 bp, and the resulting fragments underwent end polishing, A-tailing, and ligation with the Illumina full-length adapter. Subsequent PCR amplification was carried out. After purification using the AMPure XP system (Beckman Coulter, USA), DNA concentration was measured using the Qubit® 3.0 fluorometer (Invitrogen, USA). Library size distribution was analyzed with the Agilent 2100 Bioanalyzer, and quantification was done by real-time PCR (> 2 nm). Index-coded samples were clustered using the cBot Cluster Generation System with the Illumina PE Cluster Kit (Illumina, USA). Following cluster generation, the DNA libraries underwent sequencing on the Illumina platform at Novogene, producing 150-bp paired-end reads. Raw data, in the form of short reads in FASTQ format, were obtained through base calling from the original fluorescence image files.

To ensure reliable downstream bioinformatics analysis, quality control measures were applied, including discarding paired reads containing adapter contamination (> 10 nucleotides aligned to the adapter with ≤ 10% mismatches), discarding reads with > 10% uncertain bases, and discarding reads with > 50% low-quality bases (Phred quality < 5) through the use of fastp [28]. Valid sequencing data were then mapped to the *S. viridis* cultivar A10 reference genome (accession number: GCA_005286985) using the Burrows-Wheeler Aligner software [29], and the original mapping results were stored in Binary Alignment Map (BAM) format (parameter: mem -t 4 -k 32 -M). Duplicate reads were removed using SAMtools (parameter: samtools rmdup) [30] and Genome Analysis Toolkit (GATK v4.1.4) [31]. Raw single nucleotide polymorphism (SNP) sets were called by SAMtools (parameters: mpileup -m 2 -F 0.002 -d 1000) [30]. These sets were filtered based on criteria such as a depth of the variate position > 4 and a mapping quality > 2. Biallelic SNPs with a missing frequency < 10% and a minor allele frequency > 0.05 were retained for phylogenetic analysis and correlation analysis. A total of 12,849,803 SNPs were initially obtained, and 3,297,733 SNPs were retained after applying quality filtering with a minimum allele frequency threshold of 0.05.

The dist() function in FactoMineR [32] was used to generate a dissimilarity distance matrix of SNP with the Euclidean distance method. The optimal number of clusters was determined as 5 through the gapstat() function within the R package factoextra [33]. For dendrogram construction, we utilized the R package dendextra [34]. The vegdist() function in the vegan package was used to generate the distance matrices of SNP data (Euclidean) and microbial community composition (bray) [35]. The mantel() function was used for the mantel test to determine the significance of Pearson’s correlation between the SNP data and the community composition [36].

### High-throughput sequencing of the *16S rRNA* gene and internal transcribed spacer (ITS) region

Amplicon libraries for bacteria and fungi were generated using a two-step PCR approach. The hypervariable V5-V7 region of the bacterial *16S rRNA* gene was amplified with the primer pair 799F (5′-AACMGGATTAGATACCCKG-3′)/1193R (5′-ACGTCATCCCCACCTTCC-3′), and the ITS1 region was amplified with primers ITS1-F (5′-CTTGGTCATTTAGAGGAAGTAA-3′)/ITS2 (5′-GCTGCGTTCTTCATCGATGC-3′) [12, 37]. Each 30-μl PCR reaction contained 15 μl 2 × Phusion® High-Fidelity PCR Master Mix (New England Biolabs), 3 μl 2 μm forward and reverse primers, and 10 μl DNA template and was adjusted to 30 μl with double-distilled H_2_O (ddH_2_O). PCR conditions were as follows: initial denaturation at 98°C for 1 min, followed by 30 cycles of denaturation at 98°C for 10 s, annealing at 50°C for 30 s, and extension at 72°C for 30 s; and a final extension step at 72°C for 5 min. The PCR products were extracted from a 2% (w/v) agarose gel after electrophoresis, then mixed and purified with a GeneJET Gel Extraction Kit (Thermo Scientific) according to the manufacturer’s instructions. Sequencing libraries were generated with the Illumina TruSeq DNA PCR-Free Library Preparation Kit (Illumina, USA) following the manufacturer’s instructions, with added index codes. Library quality was assessed using the Qubit 2.0 Fluorometer (Thermo Scientific) and Agilent Bioanalyzer 2100 system. Finally, libraries were sequenced on an Illumina NovaSeq platform at Novogene Bioinformatics Technology Co. Ltd (Beijing, China), generating 250-bp paired-end reads.

### Quality filtering and statistical analysis of sequencing data

**Figure 2:**
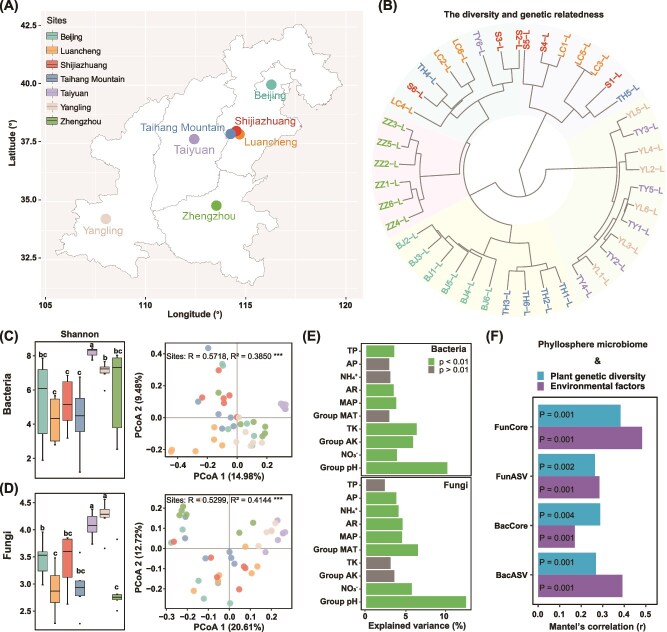
Microbial community diversity and structure of green foxtail phyllosphere microbiome affected by environmental factors and plant genetic diversity. **A**. Sampling sites in China indicated by different colors. **B**. Dendrogram displays the genetic relatedness of 42 green foxtail leaf samples used in this study based on the SNP distance (BJ, Beijing; LC, Luancheng; S, Shijiazhaung; TH, Taihang Mountain; TY, Taiyuan; YL, Yangling; ZZ, Zhengzhou). **C** and **D**. Bacterial and fungal α-diversity (Shannon index; different lowercase letters represent statistically significance, *P* < 0.05 ) and principal coordinate analysis (PCoA) based on Bray–Curtis dissimilarity with analysis of similarities (ANOSIM, *R* value) and permutational analysis of variance (PERMANOVA, *R*^2^ value) to show significant effect of sites on community composition (^***^P < 0.001). **E**. Effect of soil and climate factors or groups of collinear variables on bacterial (left) and fungal (right) community composition. Group pH includes the factors pH altitude, latitude, longitude, RH (relative humidity) and AS (accumulated sunshine duration). Group AK (available potassium) includes the factors AK, TN (total nitrogen) and SOC (soil organic carbon). Group MAT includes the factors AT (accumulated temperature) and MAT (mean annual temperature). Source data of environmental factors are provided in Supplementary Table S2. **F**. Mantel’s *r* statistic plotted for the correlation between environmental factors as well as host genetic distance and microbiome distance. BacASV and FunASV represent the microbial community distance containing all ASVs of bacteria and fungi, respectively. BacCore and FunCore denote the microbial community distance containing the core genera of bacteria and fungi, respectively.

**Figure 3:**
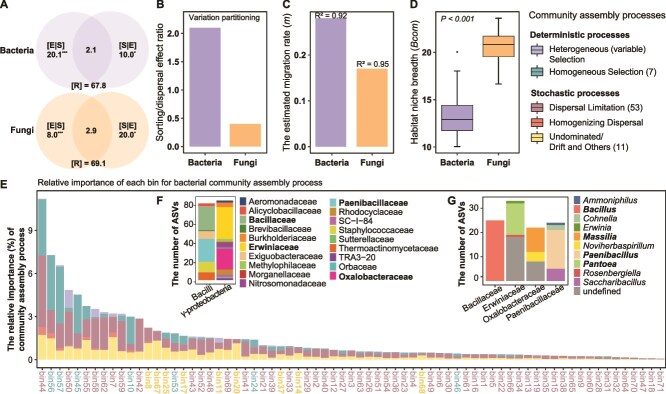
Bacterial and fungal community assembly of green foxtail phyllosphere. **A**. Variation partitioning of bacterial versus fungal communities. [E|S], [S|E] and [R] represent pure environmental fraction, pure spatial fraction and unexplained fraction, respectively. Explained variation for each component is shown in %. Asterisks indicate the statistical significance (^***^P < 0.001; ^**^P < 0.01; ^*^P < 0.05). **B**. The sorting/dispersal effect ratio (that is the ratio of [E|S] to [S|E]) obtained based on variation partitioning analysis. **C**. Fit of the neutral model. *m* indicates the estimated migration rate and *R^2^* indicates the fit to the neutral model. **D**. Mean habitat niche breadth from all taxa in each sample (*B*com) of bacterial and fungal communities. **E**. Relative importance of each bin for ecological processes represented by the different bar colors. Dominating ecological processes for each bin is displayed by the different text colors on the x-axis. **F** and **G**. Bacterial community assembly calculated via phylogenetic bin-based null model analysis. Taxonomic composition of bins (top 5) with higher relative importance of ecological processes at the family (**F**), and genus (**G**) levels.

**Figure 4:**
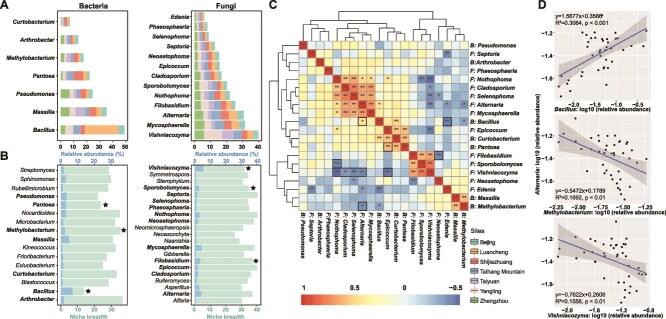
Niche breadth and correlations of core bacterial and fungal taxa in the green foxtail phyllosphere. **A**. Core bacterial and fungal genera present in all samples. Different colors represent seven sampling sites (right bottom corner). **B**. Niche breadth and average relative abundance of dominant bacterial and fungal genera. Genera with potential beneficial bacterial taxa (*Bacillus*, *Pantoea*, and *Methylobacterium*) [13, 68, 70] and yeast taxa (*Vishniacozyma*, *Filobasidium*, and *Sporobolomyces*) [57, 66] are indicated by stars. Dominant and core genera are indicated by bold fonts. **C**. Heatmaps of the correlations between the core bacterial and fungal genera. Positive and negative correlations are indicated in red and blue, respectively. **D**. Correlations of *Alternaria* and bacterial genera *Bacillus* and *Methylobacterium* as well as the yeast genus *Vishniacozyma* shown by fitting linear models.

The raw amplicon sequencing data were analyzed using QIIME 2 and the plugins [38]. DADA2 was employed to obtain amplicon sequence variants (ASVs) by removing low-quality reads, primers, denoise, and chimeras [39]. Bacterial and fungal ASVs were annotated based on the Silva (https://www.arb-silva.de/, v13.8) and UNITE (https://unite.ut.ee, v8.3) databases, respectively [40, 41]. ASVs belonging to archaea, unassigned, eukaryota, mitochondria, and chloroplast were removed. Subsequently, 1953 bacterial ASVs with 1,476,076 high-quality reads and 474 fungal ASVs with 3,041,190 high-quality reads were obtained from 42 leaf samples, respectively. The number of reads was sufficient to fully capture the bacterial and fungal diversity (Supplementary Fig. S1). Downstream analyses were performed in R (http://www.r-project.org, v4.0.3). Alpha diversity was analyzed based on the Shannon index, Richness, and Faith’s Phylogenetic Diversity (PD_whole_tree). ASV tables were normalized using the cumulative-sum scaling method prior to β-diversity analysis and subsequent analyses [42]. The Bray–Curtis distance between samples was calculated with the “vegan” package. Principal coordinates analysis (PCoA) based on the Bray–Curtis distance was performed and the significance of site on community dissimilarity was tested based on analysis of similarities (ANOSIM) and permutational multivariate analysis of variance (PERMANOVA or ADONIS) [43]. Spearman’s rank correlation (*r*  < −0.7 and *r*  > 0.7, *P*  < 0.01) was used for identifying the collinearity between environmental variables via the corr.test function (“psych” package). The correlated variables were grouped into explanatory groups. Within each group, the variable with the highest *R*^2^ in univariate analyses (the best explanatory variable) was used in the model (Fig. 2E and Supplementary Table S2) [44]. Niche breadth (*B*) refers to the range of environmental conditions and resources that a species can utilize, and it is closely linked to the organism’s (species or ASV) metabolic versatility. The community-level niche breath (*Bcom*) is the average *B* value of all taxa within a given community [45, 46]. The niche.width() function (“spaa” packages) was used to calculate the niche breadth at the community level (Fig. 3D) and of dominant genera (i.e. relative abundance >1%) (Fig. 4B). Genera present in 100% of the samples with a relative abundance > 0.1% were designated as “core genera.” The corr.test function (“psych” package) was also used for the correlation across core genera (Spearman’s rank correlation, *P *< 0.05; Fig. 4C). A nonparametric statistical test (Kruskal–Wallis test or Wilcoxon test) was used to evaluate the differences of alpha diversity and taxonomic composition among sites, as well as parametric statistical tests (i.e. pairwise *t*-tests) to assess significant differences in leaf lesion area between each pair of treatments (a total of four treatments) with the “ggpubr” package. *P* values were adjusted by using the Benjamini and Hochberg false discovery rate (FDR) test [47]. The visualization of boxplot, histogram, and scatter plot as well as heatmap were based on the “ggplot2” and “pheatmap” packages, respectively. The relative importance of species sorting and dispersal limitation was determined through variation partitioning analysis. Community variation, based on pairwise Bray–Curtis dissimilarity, was separated into environmental and spatial effects [48]. Environmental and spatial variables were selected using principal coordinates analysis of neighbor matrices and the ordiR2step() function in the “vegan” package in R [35, 46, 49]. Pure environmental variation without a spatial component ([E|S]) and pure spatial variation without an environmental component ([S|E]) represent the effects of species sorting and dispersal limitation, respectively [45, 46]. The relative importance of species sorting versus dispersal limitation was calculated as the ratio ([E|S]/[S|E]). Stochastic processes in community assembly were evaluated through a neutral community model [50], where the estimated migration rate (*m*) measures dispersal limitation. Higher *m* values indicate less dispersal limitation in microbial community assembly [50]. *R^2^* reflects the fit of the parameter based on nonlinear least-squares fitting [51]. Phylogenetic bin-based null model analysis was used to calculate bacterial community assembly processes and the relative importance of individual lineages (bins) in community assembly using the “iCAMP” package [52]. Specifically, taxa were classified into distinct groups based on phylogenetic information. Null model analysis was then applied to each bin to assess the relative importance of distinct ecological processes modulating community assembly. The iCAMP approach operates at the level of individual taxa or lineages, allowing for a more detailed modeling of community assembly processes.

### Identification and culture of bacterial and yeast isolates from green foxtail leaves

To obtain culturable bacterial and yeast taxa from leaves, leaf homogenate was prepared via grounding 200 mg leaf tissue in 1 ml sterile 10-mm MgSO_4_ buffer with two 4-mm steel balls. A serial dilution to up to 10^−5^ in 100 μl was made, and 100-μl diluted leaf homogenate was spread onto 90-mm petri dishes containing selective media. For the isolation of bacteria, R2A medium (1 l: 0.5 g yeast extract, 0.5 g peptone, 0.5 g glucose, 0.5 g soluble starch, 0.3 g sodium pyruvate, 0.024 g MgSO_4_, 0.3 g K_2_HPO_4_, 0.5 g casamino acids, and 15 g agar) was used. For the isolation of yeasts, yeast extract peptone dextrose (YPD) medium, 1 l: 20 g peptone, 10 g yeast extract, 20 g glucose, and 15 g agar with pH 3.5 and 5.5 were used. Petri dishes were incubated for 3–5 days at 25°C. Colonies with distinct morphologies were selected and transferred to fresh R2A or YPD media and subcultured until pure colonies were obtained. Pure bacterial isolates were grown in LB broth (1 l: 5 g yeast extract, 10 g tryptone, and 10 g NaCl) and stored in 40% glycerol at −80°C. Pure yeast isolates were grown in YPD broth for 1–2 d and stored in 15% glycerol (v/v) at −80°C. A total of 162 bacterial isolates and 43 yeast isolates were selected, based on colony morphology, and Sanger sequenced for taxonomical identification. *16S rRNA* genes of the bacterial isolates and ITS region of yeast isolates were amplified using the universal primers 27F (5′-AGAGTTTGATCMTGGCTCAG-3′) and 1492R (5′-CGGTTACCTTGTTACGACTT-3′) and ITS1 (5′- GTTTCCGTAGGTGAACTTGC-3′) [53] and ITS4 (5′-TCCTCCGCTTATTGATATGC-3′) [54], respectively. For bacterial isolates, a 50-μl PCR mixture system including 25 μl 2 × T5 Super PCR Mix (Colony) (Beijing Tsingke Biotechnology Co., Ltd.), 2 μl of 10-μm forward and reverse primer, 2 μl culture and 19 μl ddH_2_O. The following PCR conditions were used for bacterial *16S rRNA* gene amplification: initial denaturation at 98°C for 10 min; 30 cycles of denaturation (10 s at 98°C), annealing (15 s at 56°C), and extension (50 s at 72°C); and a final extension at 72°C for 5 min.

A yeast colony rapid detection kit (CAT:RY8001; Nanjing Ruiyuan Biotechnology Co., Ltd.) was used for PCR of the yeast colony isolates. Briefly, the yeast colony was picked into a PCR tube filled with 3.5 μl yeast lysis buffer and after mixing was heated at 98°C for 10–20 min to obtain a template of the PCR. A 50-μl PCR mixture system including 25 μl 2× yeast PCR mix, 2 μl 10-μm forward and reverse primer, 1 μl template and 20 μl ddH_2_O. The following PCR conditions were used for bacterial *16S rRNA* gene amplification: initial denaturation at 98°C for 10 min; 30 cycles of denaturation (10 s at 98°C), annealing (15 s at 56°C), and extension (50 s at 72°C), and a final extension at 72°C for 5 min. The PCR products were purified with a GeneClean Turbo kit (Qbiogene Inc., Irvine, CA) according to the manufacturer’s suggested protocol. The PCR products were used for Sanger sequencing (Beijing Tsingke Biotechnology Co., Ltd.). To identify unique isolates, BOX-PCR was performed using the primers BOX-A1R (5′-CTACGGCAAGGCGACGCTGACG-3′) [55].

### Antagonistic activities against *A. alternata* on leaves

To verify that SynComs can effectively protect plants against the foliar pathogen *A. alternata* BXL, three SynComs were designed and included: 1) core bacterial isolates (BacSynCom: (16 *Bacillus*, 6 *Pantoea*, and 1 *Methylobacterium* isolates), 2) core yeast isolates (YeastSynCom: 13 *Filobasidium*, 8 *Vishniacozyma*, and 2 *Sporobolomyces* isolates), and 3) core bacterial and yeast isolates (BacYeastSynCom: all 46 isolates from SynComs 1 and 2). These SynComs were used for the detached leaf assays. Seeds of the foxtail millet cultivar C31 were surface sterilized by soaking in 8% sodium hypochlorite for 10 min and rinsed with sterile water five times. Sterilized seeds were grown to the 2-leaf stage in sterile vermiculite under controlled conditions at 25°C with a 16 h light/8 h dark photoperiod and 60% relative humidity. The seedlings were then transplanted into pots (210 × 160 mm) filled with peat (Kleisman, Germany) and grown to the 5-leaf stage. Leaf sections with 8-cm length from the 5th leaf were excised and placed in a square Petri dish (90 × 90 mm) containing 1.5% plant agar with pH 7 [56]. Two wounds were punctured in the center of each leaf section with a sterile toothpick. Bacterial and yeast isolates were cultured in LB and YPD broth overnight at 25°C at 180 rpm, respectively. Cells were washed using water three times by centrifugation at 5,000 rpm for 5 min and then resuspended in 0.025% Tween 20. Isolates with the same OD_600nm_ value were mixed in equal volume ratios to obtain the same OD_600nm_ values for each isolate in the synthetic community (the concentration of the synthetic community was the OD_600 nm_ value of each isolate × the number of isolates). How the OD_600nm_ values translated into actual CFUs was not determined for the total of 46 SynCom members (23 bacterial and 23 yeast isolates) but there may have been introduced differences in actual densities of the initial inoculum of members of the different SynComs. Subsequently, 5-μl suspensions with the SynComs (OD_600nm_ = 0.01 per isolate) were applied on the wounds. The negative group received 5 μl 0.025% Tween 20. After 48 h, 5 μl *A. alternata* BXL at concentrations of 10^7^ spores/ml was applied at the same spot. To maintain high humidity, 2 ml sterile water was added to each plate. The plates were sealed and incubated in a growth chamber for 7 days at 25°C under a light cycle of 16 hours light/8 hours dark, following the protocol reported by Perochon and Doohan (2016) [56].

### Antifungal activity via *in vitro* dual culture assay

Antagonistic activities of the bacterial and yeast strains against the fungal pathogens *A. alternata* (BXL, DJQ), *Epicoccum nigrum* (ACCC37858), and *Pyricularia grisea* (hlj-2) were assessed as previously described by Gouka *et al.*, (2022) [57]. BXL (isolated from *Astragalus membranaceus* roots with root rot disease), DJQ (isolated from *Nicotiana tabacum* leaves with brown spot), ACCC37858 (purchased from Agricultural Culture Collection of China), and hlj-2 (isolated from *S. italica* leaf with blast disease) were incubated on PDA plates at 25°C for 9, 15, 20, and 10 d, respectively. Briefly, a mycelial plug (diameter 5 mm) was placed at the center of a 90-mm petri dish. Four 2.5-μl droplets of microbial solution (OD_600nm_ = 0.5) were spotted symmetrically at four points away from the mycelial plug 2.5 cm, respectively. A volume of 2.5 μl of LB (for bacterial isolates) and YPD (for yeast isolates) media was used in the controls. Petri dishes were incubated at 25°C. Inhibition percentage of mycelium growth was calculated as [(area_control_-area_treatment_)^*^100/area_control_].

## Results

### Genetic variation of wild green foxtail plants in native environments

We sampled leaves of wild green foxtail *S. viridis* populations at the heading developmental stage at seven sites from the center of origin in China (Fig. 1A and2A). The wild green foxtail samples clustered with *S. viridis* or *S. italica* and were distinct from *S. adhaerens*, *S. grisebachii*, and *Cenchrus americanus*, based on the phylogenetic analyses of concatenated *5S rRNA* and *knotted1* (*kn1*) genes (Supplementary Fig. S2). SNP analysis was used to further assess the genetic relatedness among the wild green foxtail samples from the seven sampling sites. Hierarchical clustering revealed five major groups, with samples not entirely clustered by site (Fig. 2B). Samples from Zhengzhou (ZZ1–6) formed one cluster, samples from Taiyuan (TY1–5) and Yangling (YL1–6) constituted a second cluster, and those samples from Beijing (BJ1–6) and Taihang Mountain (TH1–3, TH6) formed a third cluster. Samples from Luancheng and Shijiazhuang were generally distributed over two clusters (Fig. 2B).

### Effects of environmental factors and climate factors on the phyllosphere microbiota of wild green foxtail

To assess the effects of environmental and climatic factors on the phyllosphere microbiota, leaves from wild green foxtail plants (total of 42 from 7 distinct geographic locations) were subjected to amplicon sequencing of bacterial and fungal communities. We found significant differences in alpha diversity across sites (Fig. 2C and2D and Supplementary Fig. S3) and spatial patterns in bacterial ANOSIM: *R* = 0.5718, *P* < 0.001; PERMANOVA: *R*^2^ = 0.3850, *P* < 0.001; and fungal ANOSIM: *R* = 0.5299, *P* < 0.001; and PERMANOVA: *R*^2^ = 0.4144, *P* < 0.001 beta-diversity (Fig. 2C–D). Noncolinear environmental factors (i.e. pH, ${\text{NO}_{3}}^{-}$−N, ${\text{NH}_{4}}^{+}$−N, AK, TK, MAT, MAP, AR, AP, and TP) correlated with variations in bacterial and fungal community composition across sites. For bacterial communities, pH explained the largest variance (*R*^2^ = 0.10, *P* < 0.01), followed by TK (*R*^2^ = 0.064), AK (*R*^2^ = 0.059), ${\text{NO}_{3}}^{-}$–N (*R*^2^ = 0.039), MAP (*R*^2^ = 0.038), TP (*R*^2^ = 0.035), and AR (*R*^2^ = 0.035), collectively explaining 37.2% bacterial community variation (Fig. 2E and SI and Appendix Table S2). For the fungal community, pH again accounted for the largest variance (*R*^2^ = 0.126), followed by MAT (*R*^2^ = 0.065), ${\text{NO}_{3}}^{-}$–N (*R*^2^ = 0.057), AR (*R*^2^ = 0.046), MAP (*R*^2^ = 0.045), ${\text{NH}_{4}}^{+}$–N (*R*^2^ = 0.041) and AP (*R*^2^ = 0.038), with these factors together explaining 41.8% of fungal community variation (Fig. 2E and Supplementary Table S2). In addition, a significant positive correlation was observed between environmental factors and the distance in both bacterial (all ASVs: Mantel *r* = 0.3914, *P* = 0.001; core genera: *r* = 0.1705, *P* = 0.001) and fungal (all ASVs: Mantel *r* = 0.2847, *P* = 0.002; core genera: *r* = 0.4826, *P* = 0.001) communities (Fig. 2F). The bacterial and fungal communities in the phyllosphere of wild green foxtail significantly correlated with host genetic distance (all ASVs: bacterial Mantel *r* = 0.2687, *P* = 0.001; core genera: *r* = 0.2881, *P* = 0.004; fungal Mantel *r* = 0.2637, *P* = 0.002; core genera: *r* = 0.3826, *P* = 0.001) (Fig. 2F). Further tanglegram analyses supported a positive correlation between host genetic distances and phyllosphere microbiota variations, with entanglement values of 0.5 for bacterial community (Supplementary Fig. S4A) and 0.44 for fungal community (Supplementary Fig. S4B).

### Modeling ecological processes structuring the wild green foxtail phyllosphere microbiota

We quantified the interplay of distinct ecological processes structuring the assembly of the green foxtail phyllosphere microbiota using null modeling analysis. In brief, the ratio of species sorting to dispersal limitation effect (SDER) was calculated to determine the relative importance of each process. The results revealed a higher SDER for bacterial communities than for fungal communities (Fig. 3A). Bacterial and fungal communities well fitted the null model distributions (*R*^2^ = 0.92 and *R*^2^ = 0.95), with higher migration rates (*m*) in bacterial (0.28) than in fungal communities (0.17) (Fig. 3C), suggesting a relatively lower effect of dispersal limitation in bacterial communities. The community-level habitat niche breadth (*Bcom*) was significantly lower in bacterial communities compared to fungal communities (Fig. 3D). Null model analysis based on phylogenetic bins and community β-diversity was performed to determine the relative importance of different ecological processes operating at the individual bacterial lineages (bins) (Fig. 3E–G). For that, bacterial ASVs were categorized into 71 groups (bins) based on their phylogenetic relationships, with 7 bins mostly structured by homogeneous selection, 53 bins by dispersal limitation, and 11 bins by ecological drift and other processes. Specific bins, including bin 44, 56, 57, 50, 45, 55, 58, 62, 7, and 59, were taxonomically assigned to 46 families, contributing ~50% to the total community (Fig. 3F and Supplementary Fig. S5). These bins mainly belonged to Erwiniaceae, Pseudomonadaceae, Bacillaceae, Paenibacillaceae, Oxalobacteraceae, Azospirillaceae, Xanthomonadaceae, Enterobacteriaceae, Micrococcaceae, Staphylococcaceae, and so on (Supplementary Fig. S5). Major bacterial genera from these bins were *Pseudomonas*, *Bacillus*, *Skermanella*, *Paenibacillus*, *Pantoea, Massilia*, *Exiguobacterium*, *Staphylococcus*, *Deinococcus*, and *Lysobacter* (Fig. 3G and Supplementary Fig. S6). Most of these families and genera were major taxa in top-5 bins (Fig. 3F and 3G). This method was not suitable for the fungal communities because the ITS region is a noncoding region with a high mutation rate, with mostly random mutations, which do not reflect the phylogenetic relationships between species.

### Defining the wild green foxtail core phyllosphere microbiota

The green foxtail phyllosphere core microbiota consists of genera detected in 100% of the leaf samples with a relative abundance higher than 0.1% (Fig. 4A and 4B and Supplementary Fig. S7). The core bacterial taxa included members of the genera *Bacillus*, *Massilia*, *Pseudomonas*, *Pantoea*, *Methylobacterium*, *Arthrobacter*, and *Curtobacterium*, whereas the core fungal taxa included three yeast genera, *Vishniacozyma*, *Filobasidium*, and *Sporobolomyces*, and the putative fungal pathogens *Alternaria*, *Nothophoma*, *Epicoccum*, and *Neosetophoma* (Fig. 4A). Individual-level niche breadth was calculated to represent the extent of individual distribution along the sampled geographical scale (i.e. higher values indicate broader habitat niche breadth). Overall, the niche breadth of these core taxa ranged from 13.89 to 36.64 for bacteria and from 25.82 to 40.55 for fungi. Members affiliated with *Pantoea* and *Methylobacterium* had wider niche breadths than the most abundant core bacterial taxa *Bacillus*. Similarly, members affiliated with *Filobasidium*, *Sporobolomyces*, *Alternaria*, *Nothophoma*, *Epicoccum*, and *Neosetophoma* had broader niche breadths than the most abundant core yeast taxa *Vishniacozyma* (Fig. 4B). Furthermore, several bacterial (*Bacillus*, *Pantoea*, and *Methylobacterium*) and yeast taxa (*Vishniacozyma*, *Filobasidium*, and *Sporobolomyces*) positively or negatively correlated with the abundances of one or more putative plant pathogenic fungal genera (*Alternaria*, *Nothophoma*, *Epicoccum*, and *Neosetophoma*) (Fig. 4C and 4D and Supplementary Fig. S8).

### Design of SynComs with potential antagonistic effects on foliar fungal pathogens

**Figure 5:**
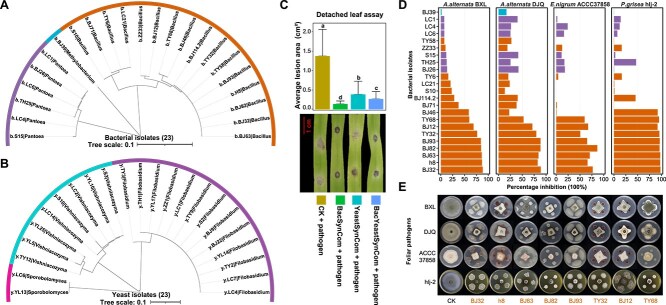
Plant protection against *A. alternata* SynComs with phyllosphere bacterial and yeast isolates. **A** and **B**. Phylogenetic tree of 23 bacterial isolates (based on *16S rRNA* gene) and 23 yeast isolates (based on ITS region) selected for the detached leaf assay, was generated with qiime and viewed in iTOL. Different colors represent different genera. **C**. Disease lesion area on leaves inoculated with different SynComs and *A. alternata* BXL. Different little letters represent the statistical significance (*t*-test, *P* < 0.05). CK + pathogen: Mock-inoculation with 5-μl 0.025% Tween 20 before inoculation with pathogen BXL; BacSynCom + pathogen: inoculation with 5-μl BacSynCom before inoculation with pathogen BXL; YeastSynCom + pathogen: Inoculation with 5-μl YeastSynCom before inoculation with pathogen BXL; BacYeastSynCom + pathogen: Inoculation with 5-μl BacYeastSynCom before inoculation with pathogen BXL. **D**. The percentage inhibition of 23 bacterial isolates against four pathogens (*A. alternata* BXL and DJQ, *E. nigrum* ACCC37858 and *P. grisea* hlj-2). The different colors of bars represent different species. **E**. Bacterial isolates with more than 50% inhibition *in vitro*.

To further investigate the potential functions of the green foxtail core phyllosphere microbiota—specifically with a focus on testing its potential to suppress the fungal leaf pathogen *A. alternata*—we obtained ~400 bacterial and yeast isolates from wild green foxtail leaves (Fig. 1B and Supplementary Figs. S9 and S10). Using a subset of 162 bacterial and 43 yeast isolates (Supplementary Fig. S9 and S10), *16S rRNA* gene and ITS amplicon sequencing allowed taxonomic assignment into 28 bacterial genera within the phyla Actinobacteriota (75), Firmicutes (69), and Proteobacteria (18), and 11 yeast genera within the phyla Ascomycota (2) and Basidiomycota (41). The design of SynComs was based on two criteria: (i) the isolate must be a core taxon present in all wild green foxtail leaf samples, and (ii) the relative abundance of this core taxon must have had a significant positive or negative correlation with the relative abundance of one or more of the putative foliar fungal pathogens. Following these criteria, BOX-PCR and dRep analysis were performed to resolve intraspecific diversity and to de-replicate the isolate collection (Supplementary Figs. S11 and S12). After that, a total of 23 bacterial isolates (*Bacillus, Pantoea, Methylobacterium*) and 23 yeast isolates (*Vishniacozyma, Sporobolomyces, Filobasidium*) were used for the design of three distinct SynComs: one SynCom containing only bacterial isolates, one SynCom containing only yeast isolates, and one cross-kingdom SynCom containing bacterial and yeast isolates (Fig. 1B, Fig. 5A-B). Next, we employed a detached leaf assay to test the effect of the designed SynComs on the infection of leaves of the domesticated foxtail millet by the foliar fungal pathogen *A. alternata*. The results revealed a significant reduction in lesion size by all three SynComs when compared to the control, i.e. mock(buffer)-inoculated leaves (CK + pathogen; average lesion area: 0.69 cm^2^). The bacterial SynCom (BacSynCom) displayed the strongest inhibition (average lesion area: 0.07 cm^2^), followed by the mixed bacterial and yeast SynCom (BacYeastSynCom; 0.14 cm^2^) and the SynCom composed of yeast isolates (YeastSynCom; 0.20 cm^2^) (Fig. 5C). Collectively, these results showed that core microbiome members of leaves of wild green foxtail sampled at geographically diverse sites in its center of origin can provide protection to domesticated foxtail millet.

## Discussion

**Figure 6:**
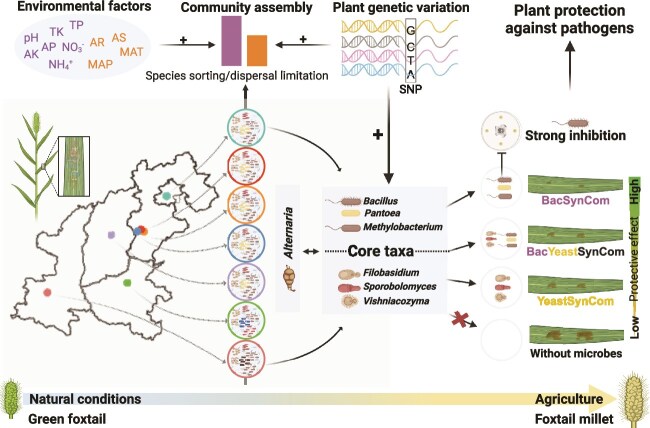
Conceptual framework depicting community assembly of green foxtail phyllosphere affected by environmental factors and plant genetic variation, as well as the plant protection of dominant core taxa against plant pathogen *Alternaria alternata* in foxtail millet. Environmental factors including soil properties (pH, TP, TK, AP, AK, ${\text{NH}_{4}}^{+}$ and ${\text{NO}_{3}}^{-}$) and climate factors (AR, AS, MAP, and MAT) and plant genetic diversity based on SNP affected the assembly of the phyllosphere microbiome from seven sites. Species sorting and dispersal limitation differently governed bacterial and fungal community assembly. Three bacterial and three yeast genera were identified as core taxa with increased correlation with plant genetic diversity compared to the whole community, which were used in dual culture assay and detached leaf assay. SynComs composed of bacterial isolates (BacSynCom), yeast isolates (YeastSynCom) and both of them (BacYeastSynCom) significantly reduced the lesion area caused by plant pathogen *A. alternata*. BacSynCom exhibited the strongest protective effect (smallest lesion area) than other two SynComs. *Bacillus*, members of SynCom, had a strong inhibitory effect via dual culture assay. Back to natural conditions to explore the potential of the wild crop ancestors’ phyllosphere microbiome in enhancing the resilience and fitness of domesticated crop counterpart.

The study of plant-associated microbiota of wild progenitors of modern crops can contribute to a better understanding of ecological and evolutionary aspects of host–microbe interactions, which constitutes an essential step toward harnessing beneficial microbiomes of wild relatives for modern day crops [1]. In addition to stochastic processes, environmental and host-driven selection are key factors structuring the diversity and functioning of plant-associated microbiota [15, 19, 58]. In this study, we show how the phyllosphere microbiota composition (bacterial and fungal communities) of the wild green foxtail in its center of origin varies as a function of geographic distances and edaphic factors at distinct localities. This finding confirms and extends results of previous studies showing that host genotype or phylogeny, as well as abiotic factors, such as soil properties, climate, and topography, significantly affect community variation in phyllosphere bacteria, fungi and oomycetes [15, 19]. The influence of location (site) on the phyllosphere microbiota has been often linked to variations in latitude, climate factors (annual temperature), soil properties (specifically nutrient content), and neighboring plant diversity [19]. Here, we show that soil edaphic properties (e.g. specifically soil pH and ${\text{NO}_{3}}^{-}$-N content) and climatic factors (e.g. MAP, RA) greatly explain the variation in the phyllosphere microbiota of wild green foxtail species (Fig. 1D). Interestingly, our analyses allowed for the integration of host genetic distances and variations in the phyllosphere microbiota. A similar approach has been applied to the rhizosphere of sorghum grown in soils from its center of origin, where plant genetic diversity across 12 sorghum genotypes significantly correlated with the rhizobacterial community composition [36]. In brief, consistent with the findings by He *et al*., (2024), a strong correlation was observed between plant genetic and microbiome distance matrices across genotypes for both root and rhizosphere communities. Under stress conditions, such as low phosphorus, nitrogen, and drought, the influence of host genetics on rhizobacterial community variation is amplified [58]. This underscores the observation that host plants adapt their root and rhizosphere microbiomes to local conditions through specific genetic traits, enhancing resilience to environmental stress. In our study, we observed distinct phyllosphere microbial communities in wild green foxtail plants grown across large geographical scales with variable environmental conditions (Fig. 6).

By using null modeling analysis, we also showed the phyllosphere microbiota to be structured by an interplay of species sorting (determined by environmental variables and host genetics) and dispersal limitation. Species sorting and dispersal limitation are affected by factors such as propagule size, metabolic activity, and distinct mechanisms of passive and active dispersal [59, 60]. A higher species sorting/dispersal effect ratio (SDER) was observed for bacterial communities in the phyllosphere (Fig. 3A-B), which indicates greater environmental constraints and lower dispersal limitation comparatively to the fungal communities. Lower dispersal limitation of the bacterial community was supported by the estimated migration rate from the neutral model (Fig. 3C). The higher SDER in the bacterial community was also reflected in the significant lower community-level habitat niche breadth (*Bcom*) (Fig. 3D), as microbial groups with narrower niche breadths generally demonstrate less metabolic flexibility and adaptability to environmental conditions at the community level [45]. These results align with the size-plasticity and size-dispersal hypotheses stating that microorganisms with lower metabolic versatility are more sensitive to ecological selection and that microorganisms with smaller body sizes have greater dispersal ability [45].

The core microbiota represents taxa consistently found in association with a host species and broader environmental conditions. One facet includes the idea of harnessing these core taxa to enhance plant fitness and/or protection against biotic and abiotic stresses [61, 62]. Zhou *et al*. (2024) revealed that SynComs composed of the native core rhizoplane microbiome can efficiently promote tobacco growth [63]. We identified several core bacterial and yeast genera in the phyllosphere of wild foxtail millet based on prevalence (i.e. present in 100% of the samples) and abundance (i.e. relative abundance > 0.1%) (Fig. 4). Specifically, isolates taxonomically assigned as *Pseudomonas*, *Sphingomonas,* and *Methylobacterium* were commonly found in the green foxtail phyllosphere. This finding confirms and extends results of previous studies of core taxa in several other plant species [9, 10, 13, 15, 18–20]. For instance, a recent study showed that *Pantoea* (a genus also detected as core in our study) in the phyllosphere of citrus contributes to plant protection against the fungal pathogen *Diaporthe citri* [13]. Interestingly, members within the genus *Bacillus* are typically detected at low abundance in the phyllosphere of *Arabidopsis* [64, 65] and are less frequently reported in other phyllosphere studies [9, 10, 13, 15, 18–20]. In our study, however, several *Bacillus* ASVs were detected as core taxa in the green foxtail phyllosphere (Fig. 4A and 4B). As for fungal taxa, the green foxtail phyllosphere core was composed of members affiliated with *Alternaria*, *Epicoccum*, and *Cladosporium.* Additionally, yeast taxa included isolates affiliated with *Vishniacozyma*, *Filobasidium,* and *Sporobolomyces*, all of which were present at high relative abundance (Fig. 4A and 4B, Fig. S6B). Recently, the significant ecological roles and functional potential of the phyllosphere yeast have been outlined by Gouka *et al*. (2022) [66]. In our study, core yeast genera (*Vishniacozyma*, *Filobasidium,* and *Sporobolomyces*) exhibited significant negative correlations with one or multiple potential foliar pathogens (*Alternaria, Epicoccum, Nothophoma, Cladosporium*, *and Selenophoma*) (Fig. 4C and S7). This information was used to further test their potential in cross-kingdom SynComs mediating antagonistic interactions in suppressing these foliar pathogens.

In this study, we designed and experimentally tested the suppressive potential of three SynComs comprising bacterial and yeast isolates by using a detached leaf assay. The design of these SynComs was informed by correlational data obtained between individual core taxa and putative fungal leaf pathogens. This strategy was effective as demonstrated by all three SynComs composed of bacterial and/or yeast isolates being found to effectively reduce the lesion area caused by the fungal pathogen *A. alternata* (Fig. 5C). Although the bacteria+yeast SynCom significantly reduced leaf infection by *A. alternata*, the SynCom composed only of bacterial isolates was the most effective in reducing fungal infection. Further assessment of the *in vitro* activity of members of these SynComs revealed strong hyphal growth inhibition of *A. alternata* by the *Bacillus* isolates (*Bacillus subtilis* BJ32, h8, BJ63, and TY32; *Bacillus velezensis* BJ82 and BJ93; *Bacillus paralicheniformis* BJ12; and *Bacillus safensis* TY68) (Fig. 5D-E). The inhibitory effects of the *Bacilli* were also significant in suppressing hyphal growth of other foliar pathogens including *Epicoccum nigrum* ACCC37858, and *Pyricularia grisea* hlj-2 (Fig. 5D-E). Although no so-called drop-out assays, where each of the members of the SynCom are omitted one by one, were performed, considering the magnitude of the SynCom composition, these *in vitro* results may suggest that the most effective members of the SynCom are the *Bacilli* who are well known for the production of specific secondary metabolites with antifungal activity [67, 68]. However, other modes of antagonism such as competition for space or nutrients may also play a role, but these are not captured in the dual confrontation assays performed here. Schäfer *et al*. (2023) have revealed that bacterial strains with significant niche overlap exhibit strong resource competition, and in coculture, strains of lower abundance demonstrate lower nutrient uptake rates than more abundant strains [69]. For the yeasts, the negative correlations between core genera abundance and foliar pathogen relative abundance suggest density-dependent suppression mechanisms, likely involving resource competition. However, the competitive dynamics between filamentous fungi and yeasts regarding resource and space utilization remain to be elucidated.

In conclusion, our study provided fundamental insight into the mechanisms governing phyllosphere microbiota assembly of a wild crop ancestor across large geographic scales. This knowledge was highly instrumental for the design of cross-kingdom SynComs that mitigate biotic stress of a modern-day agricultural crop. Finally, the obtained results also pave the way for uncovering the natural functions of phyllosphere microbiota for the ecology and evolution of wild crop ancestors in their natural habitats.

## Supplementary Material

Supplementary_Final_ycaf066

## Data Availability

The raw sequence data (16S rRNA and ITS reads) reported in this manuscript are available in the NCBI Sequence Read Archive under BioProject PRJNA1114365.

## Code availability

R scripts used in this manuscript are available at https://github.com/Xiaoyu-sys/2024NEE_GREEN_FOXTAIL_LEAF.git.
