# Supplementary material for: Harnessing the phyllosphere microbiota of wild foxtail millet for designing beneficial cross-kingdom synthetic communities
Source: ISME Commun. 2025 May 3;5(1):ycaf066. doi: 10.1093/ismeco/ycaf066 (PMC12286919; doi:10.1093/ismeco/ycaf066)
Supplement: Supplementary_Final_ycaf066 [file supplementary_final_ycaf066.pdf]

Harnessing the phyllosphere microbiota of wild foxtail millet for designing beneficial cross-kingdom synthetic communities

Xiaoyu Zai[1,2,3,4], Feng Zhu[5], Meicheng Zhao[5,6], Xianmin Diao[6], Fusuo Zhang[1,2,3,4], Francisco Dini-Andreote[7,8], Chrats Melkonian[9,10], Marnix H. Medema[9], Jos M. Raaijmakers[11,12], Viviane Cordovez[11], Chunxu Song[1,2,3,4]✉

1 State Key Laboratory of Nutrient Use and Management, College of Resources and Environmental Sciences, China Agricultural University, Beijing, China;

2 National Academy of Agriculture Green Development, China Agricultural University, Beijing, China;

3 Key Laboratory of Plant-Soil Interactions, Ministry of Education, China Agricultural University, Beijing, China;

4 National Observation and Research Station of Agriculture Green Development, Quzhou, Hebei;

5 Key Laboratory of Agricultural Water Resources, Hebei Laboratory of Agricultural Water-Saving, Center for Agricultural Resources Research, Institute of Genetics and Developmental Biology, The Innovative Academy of Seed Design, Chinese Academy of Sciences, 050021 Shijiazhuang, China;

6 Institute of Crop Sciences, Chinese Academy of Agricultural Sciences, 100081 Beijing, China;

7 Department of Plant Science & Huck Institutes of the Life Sciences, The Pennsylvania State University, University Park, PA 16802, USA;

8 The One Health Microbiome Center, Huck Institutes of the Life Sciences, The Pennsylvania State University, University Park, PA, USA;

9 Bioinformatics Group, Wageningen University & Research, 6708 PB Wageningen, the Netherlands;

10 Theoretical Biology and Bioinformatics, Science for Life, Utrecht University, Utrecht, the Netherlands;

11 Department of Microbial Ecology, Netherlands Institute of Ecology, Wageningen, The Netherlands;

12 Institute of Biology, Leiden University, Leiden, the Netherlands

✉ e-mail: chunxu.song@cau.edu.cn ✉ e-mail: chunxu.song@cau.edu.cn

Table S1 Soil and climate factors of seven sampling sites in this study.

	Beijing	Luancheng	Shijiazhuang	Taihang Mountain	Taiyuan	Yangling	Zhengzhou
pH	8.42±0.07	9.09±0.05	9.12±0.02	9.11±0.03	8.2±0.03	8.23±0.05	8.52±0.06
NO_3^--N (mg/kg)	4.78±3.04	35.2±22.2	22.66±10.36	28.11±11.3	10.67±1.15	13.82±1.49	1.75±0.58
NH_4^+-N (mg/kg)	1.21±0.56	3.23±0.83	3.42±1.43	3.42±1.43	2.78±0.77	3.37±1.4	2.85±1.15
AK (g/kg)	172.28±56.26	227.7±25.86	235.45±46.32	205.65±74.4	283.12±3.2	184.8±5.42	85.88±1.46
AP (mg/kg)	15.69±6.26	23.65±8.95	17.37±6.04	18.73±13.39	11.34±1.11	6.79±0.86	10.07±0.22
TK (g/kg)	17.85±2.05	17.27±2.85	16.64±1.28	17.85±1.37	10.33±1.4	20.54±0.87	16.34±0.61
TP (g/kg)	0.59±0.07	0.82±0.26	0.69±0.16	0.62±0.22	0.7±0.05	0.94±0.03	0.61±0.01
TN (g/kg)	0.07±0.02	0.13±0.03	0.15±0.05	0.19±0.11	0.17±0.04	0.13±0.01	0.03±0.01
SOC (g/kg)	0.73±0.25	1.25±0.25	1.62±0.61	2.06±1.42	3.43±0.42	0.86±0.03	0.2±0
Altitude (m)	39.71±3.33	44.38±4.03	63.72±0	347.84±3.85	766.33±0	417.21±0	89.53±0
Longitude	116.18.01	114.69	114.53	114.25	112.43	108.02	113.53
Latitude	39.77.01	37.89	38.03	37.9	37.68	34.25	34.83
MAT (℃)	11.72	13.09	10.56	13.61	10.42	12.55	15.48
AT (℃)	1956.3	2223.7	1173.6	2273.1	1730.3	1966.7	2528
RH (%)	47.37	52.93	47.1	50.51	48.22	66.55	52.75
MAP (mm)	0.55	0.5	0.92	0.45	0.77	1.21	0.79
AS (h)	1161.3	1191.7	1256.6	1195.4	1106	570.3	985.7
AR (MJ/m^2)	3061.906	3180.545	3177.874	3173.428	2805.101	3038.888	3210.522

NO_3^--N, nitrate nitrogen; NH_4^+-N, ammonium nitrogen; AK, available potassium; AP, available phosphorus; TK, total potassium; TP, total phosphorus; TN, total nitrogen; SOC, soil organic carbon; MAT, mean annual temperature; AT, accumulated temperature; RH, relative humidity;

MAP, mean annual precipitation; AS, accumulated sunshine duration; AR, annual cumulative solar radiation.

Note: Data are mean ± standard deviation (n = 6).

Table S2 The Explained variance of soil and climate factors on phyllosphere microbial community assemblages based on ASVs distance matrices (Bray Curtis dissimilarities) using PERMANOVA partitioning

	Bacteria				Fungi			
	df	F	R^2	*P*	df	F	R^2	*P*
Group pH	1	5.849	**0.102**	**0.001**	1	7.93	**0.126**	**0.001**
NO_3^-	1	2.208	**0.039**	**0.003**	1	3.62	**0.057**	**0.001**
NH_4^+	1	1.736	0.03	0.026	1	2.566	**0.041**	**0.008**
Group AK	1	3.377	**0.059**	**0.001**	1	2.231	0.035	0.026
AP	1	1.651	0.029	0.02	1	2.385	**0.038**	**0.009**
TK	1	3.637	**0.064**	**0.001**	1	1.936	0.031	0.046
TP	1	2.013	**0.035**	**0.002**	1	1.479	0.023	0.116
Group MAT	1	1.661	0.029	0.025	1	4.102	**0.065**	**0.001**
MAP	1	2.156	**0.038**	**0.002**	1	2.846	**0.045**	**0.001**
AR	1	1.978	**0.035**	**0.004**	1	2.887	**0.046**	**0.002**
Residuals	31	NA	0.541	NA	31	NA	0.492	NA
Total	41	NA	1	NA	41	NA	1	NA

Group pH includes the factors pH, altitude, latitude, longitude, RH (relative humidity) and AS (accumulated sunshine duration). Group AK (available potassium) includes the factors AK, TN (total nitrogen) and SOC (soil organic carbon). Group MAT includes the factors AT (accumulated temperature) and MAT (mean annual temperature).

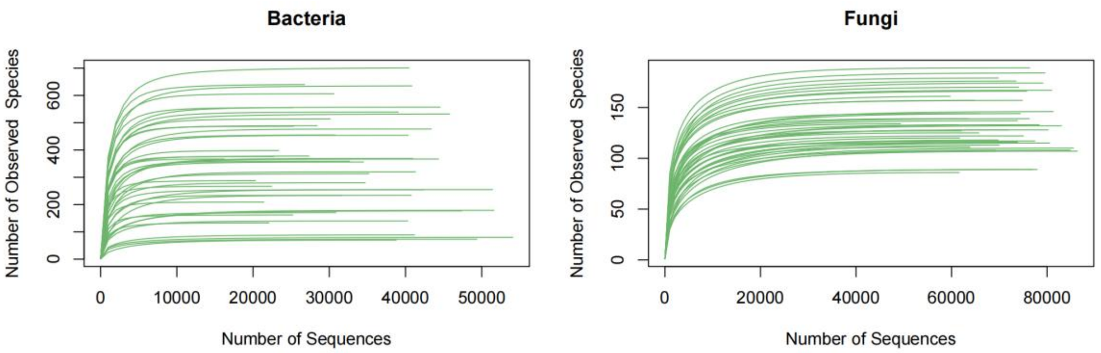

Fig. S1 Rarefaction curves of quality-controlled reads.

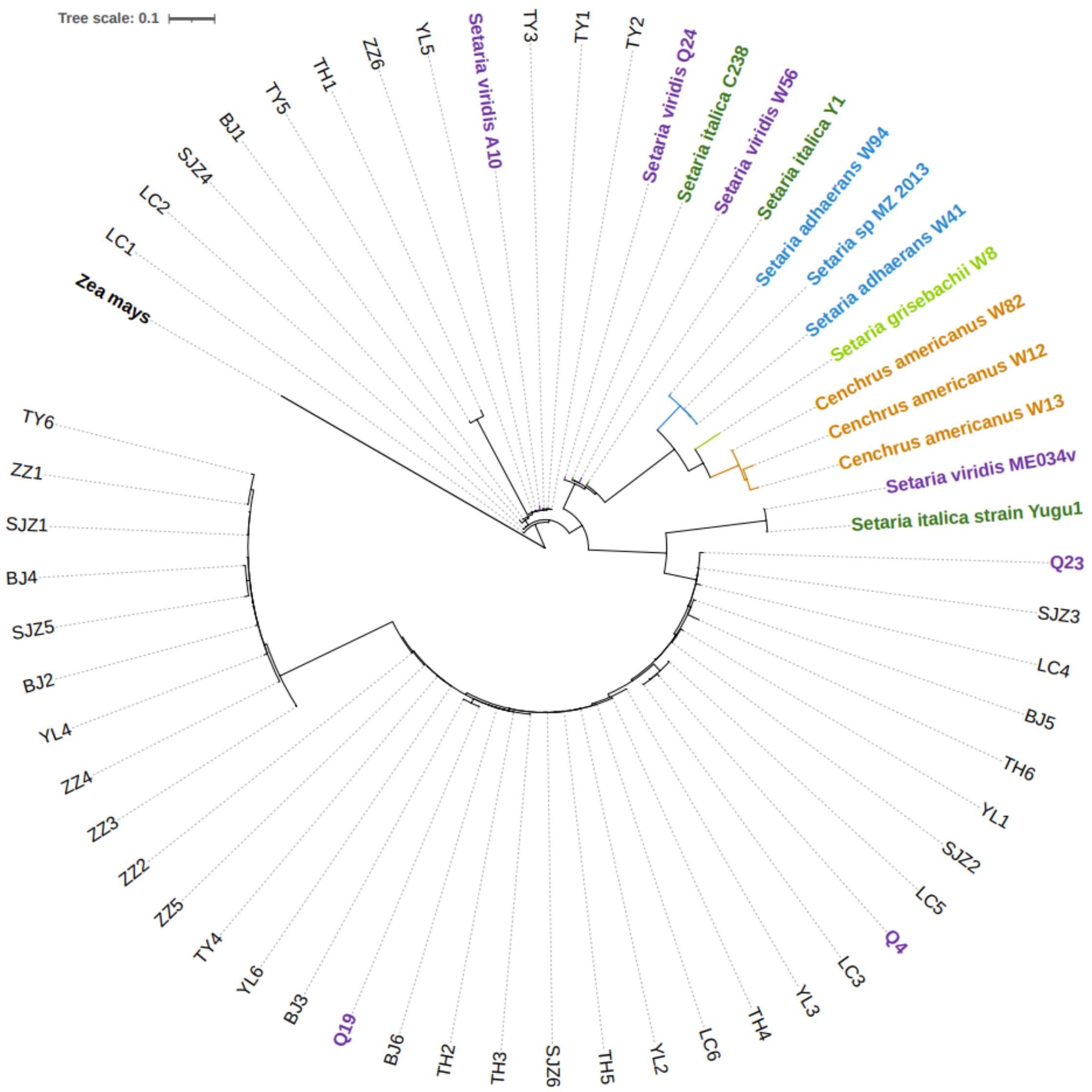

Fig. S2 Phylogeny of *Seteria* based on the concatenation of *5S rDNA* and *kn1* gene

sequences. Black fonts represent the samples utilized in this study, bold black font represent the outgroup *Zea mays*, while other colors represent different *Seteria* genera including *S. viridis* (green foxtail) colored in purple.

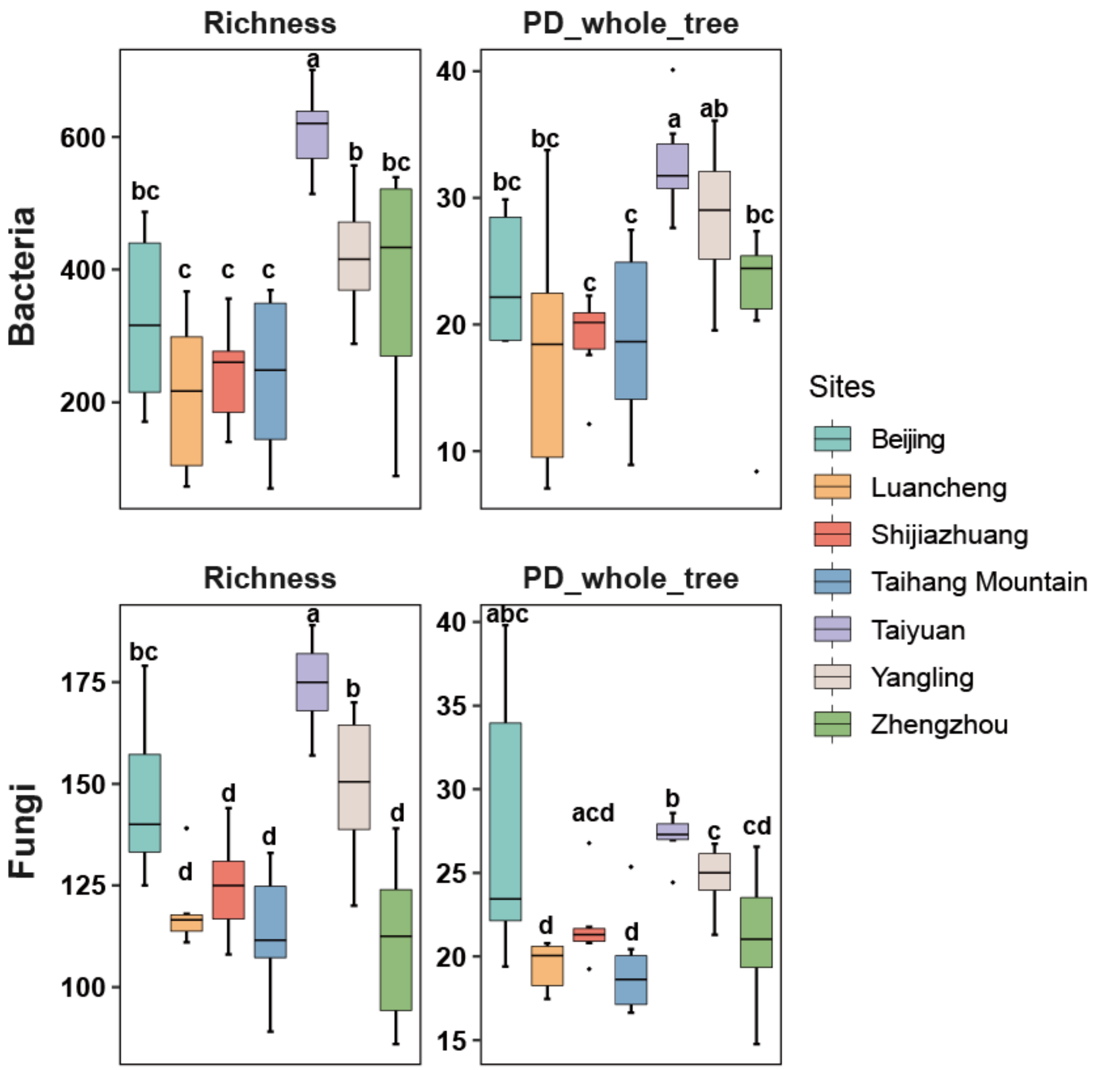

Fig. S3 The alpha diversity including richness and PD_whole_tree. Different colors represent different sites. Different lowercase letters represent statistically significance, $P < 0.05$.

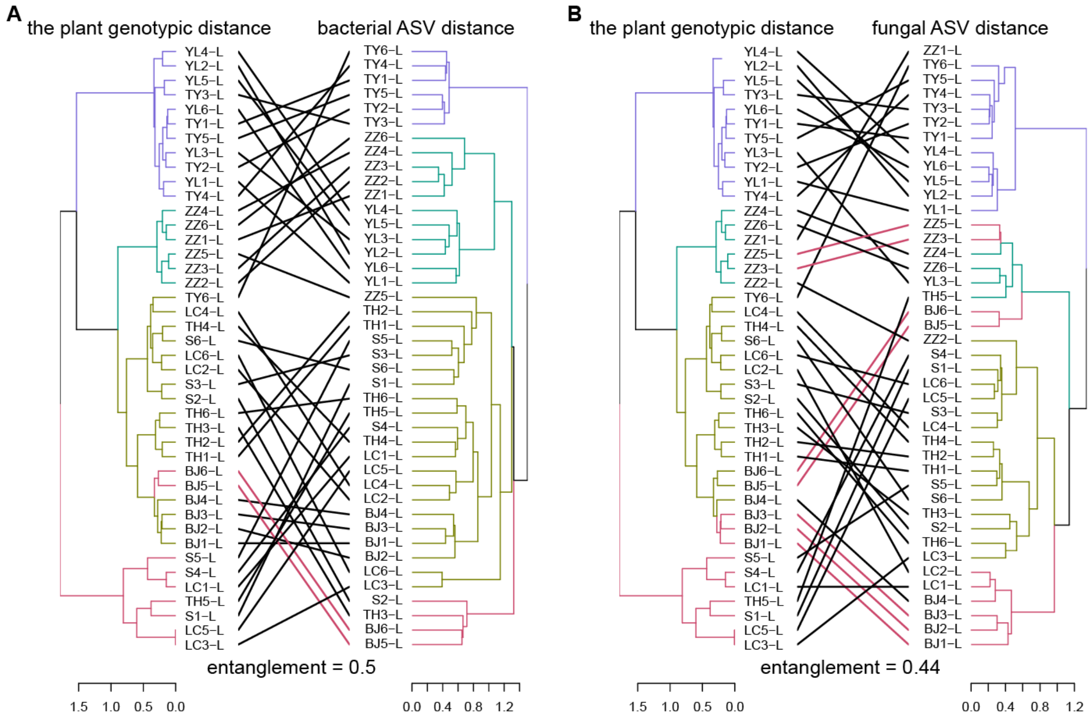

Fig. S4 Correlation between the green foxtail genetic distance and the microbial community composition. A-B Tanglegrams displaying the entanglement between the green foxtail genetic distance based on the SNP analysis and the phyllosphere microbiome based on *16S rRNA* gene (bacteria) and ITS1 region (fungi) amplicon analysis across 42 samples from seven sites.

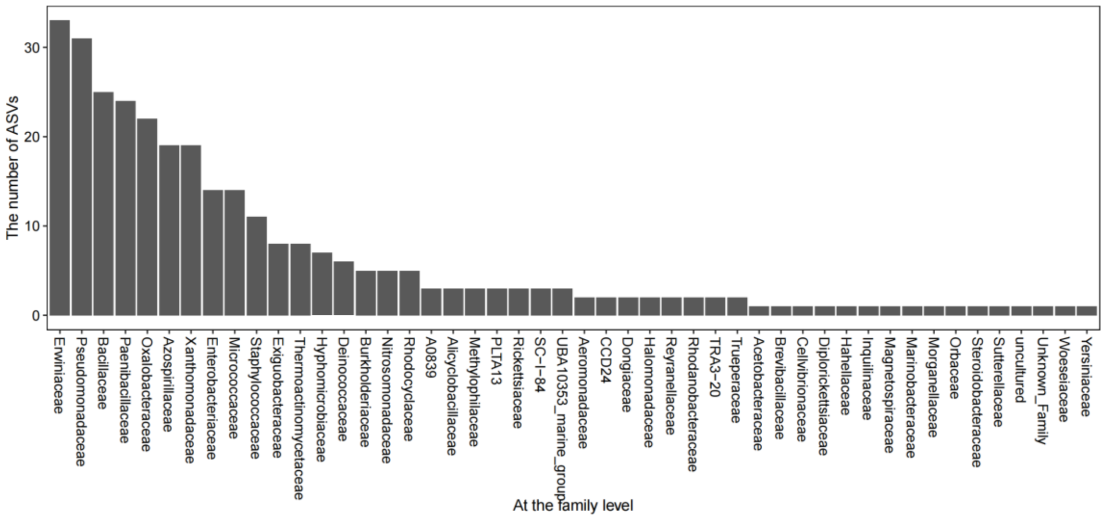

Fig. S5 The number of ASVs of the families of the top 10 bins based on the relative importance to bacterial community assembly. All bacterial ASVs were clustered into 71 groups (bins) with varied relative importance to bacterial community assembly via phylogenetic bin-based null model analysis ("iCAMP" package). The top 10 bins, based on the relative importance to bacterial community assembly, belonged to 46 families.

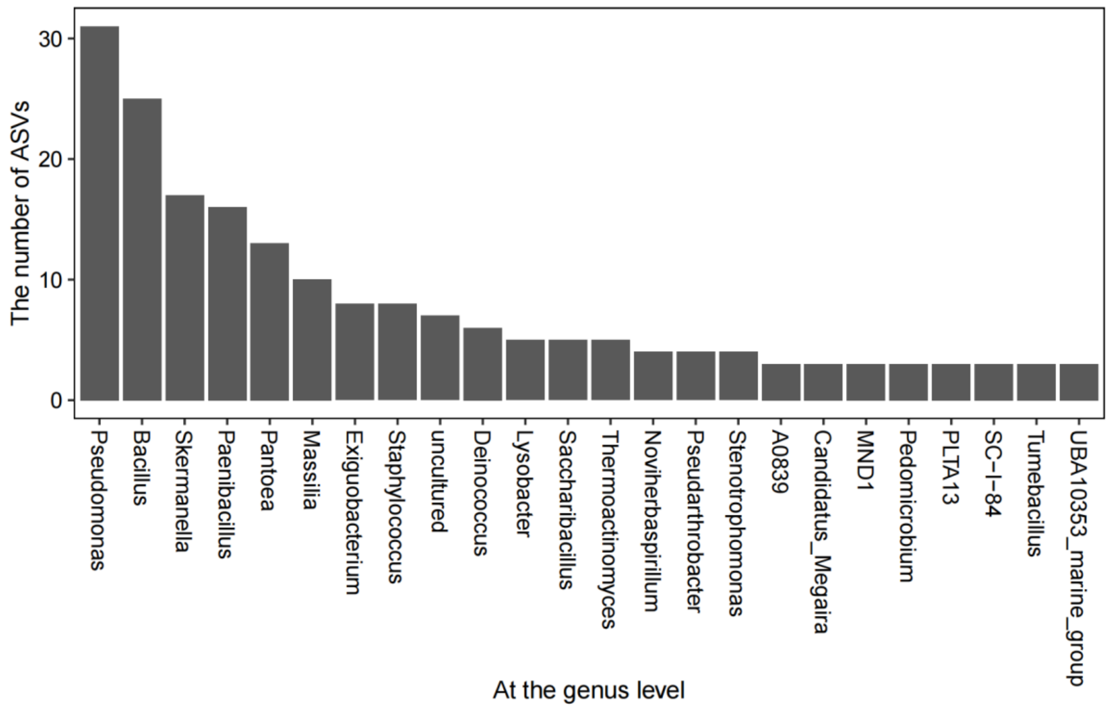

Fig. S6 The number of ASVs belonging to top 20 genera of the top 10 bins based on the relative importance to bacterial community assembly. All bacterial ASVs were clustered into 71 groups (bins) with varied relative importance to bacterial community assembly via phylogenetic bin-based null model analysis ("iCAMP" package). The top 10 bins, based on the relative importance to bacterial community assembly, belonged to 65 genera. Top 20 genera, based on the ASV numbers, were selected to be shown.

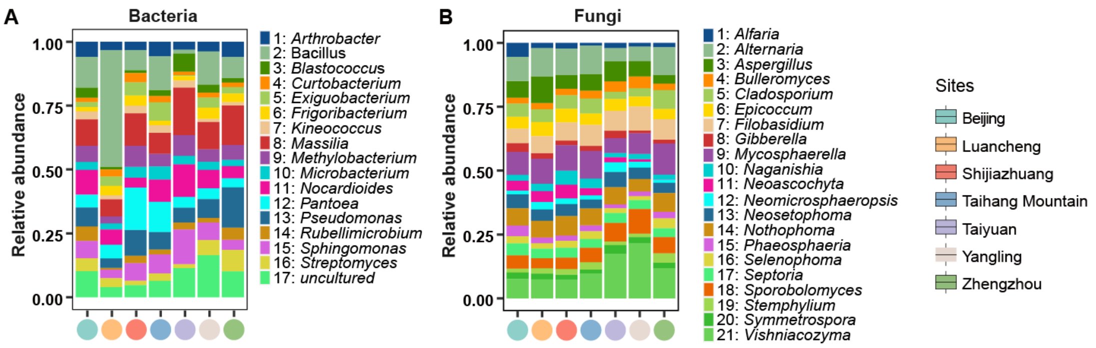

Fig. S7 A, B Dominant bacterial and fungal genera with relative abundance more than 0.01 shown by stacked bar chart. Different colors of the bar represent different genera. Points with different colors on the abscissa represent seven sites.

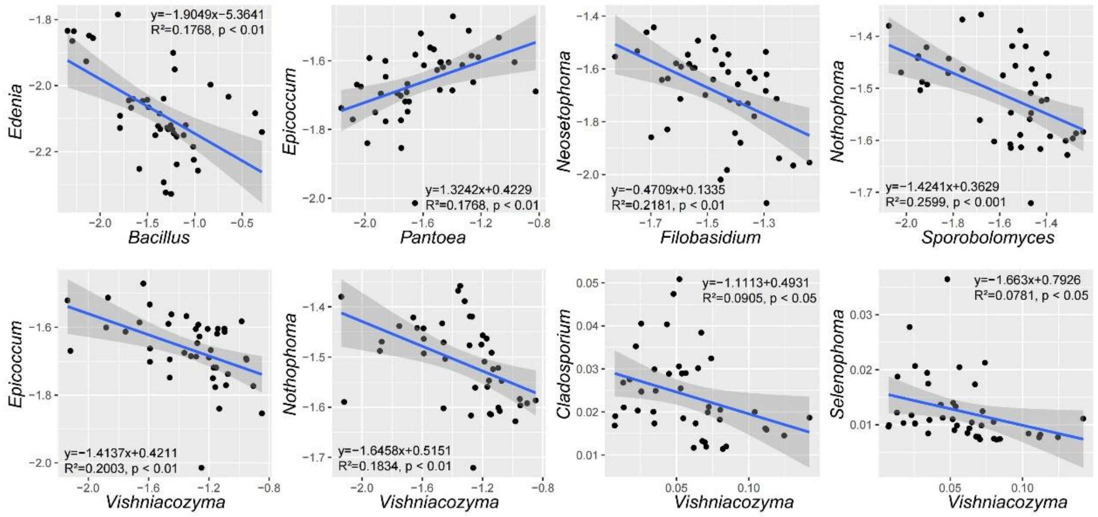

Fig. S8 The correlations between core bacterial as well as yeast genera and putative fungal pathogenic genera shown by fitting linear models.

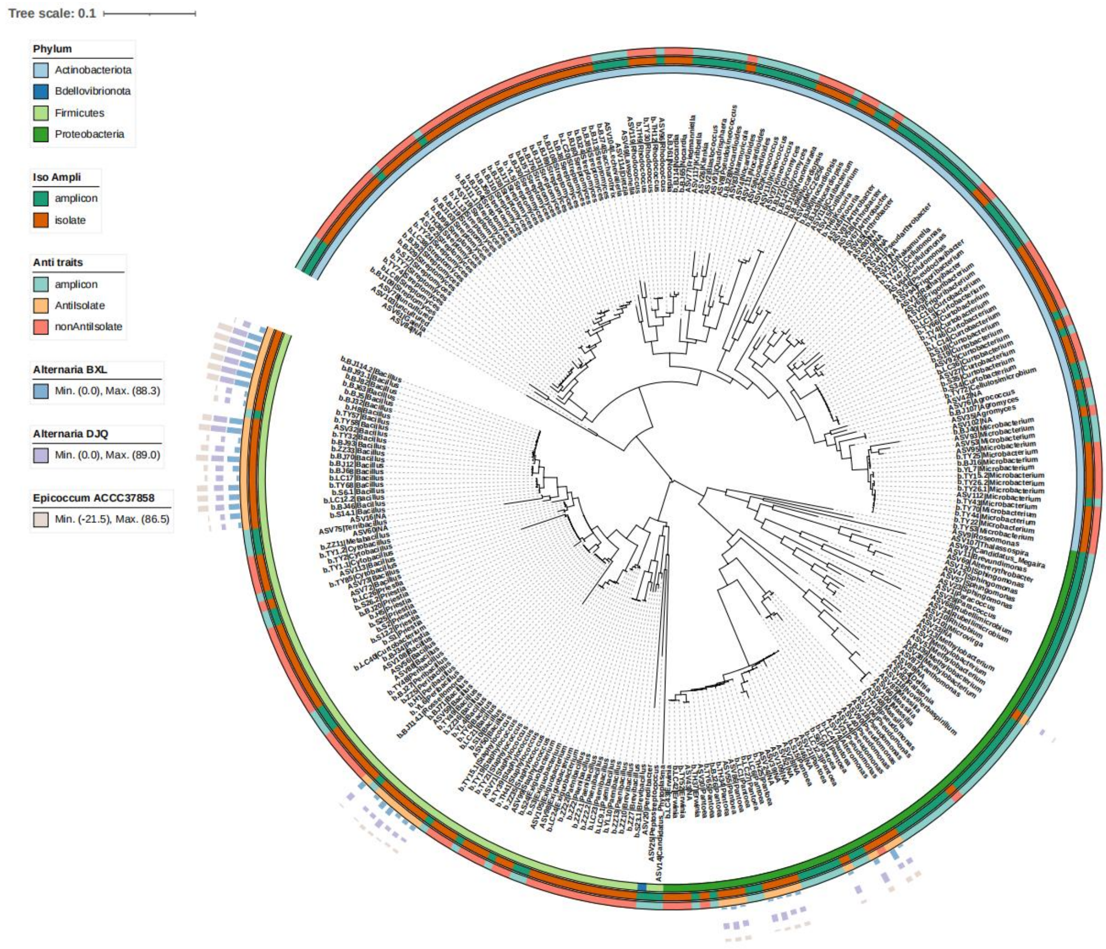

Fig. S9 Phylogenetic tree of 162 bacterial isolates and ASVs occurred in more than 50% samples based on 16S rRNA gene sequences. From the inside to the outside, the three rings represent "Phylum", "Iso Ampli", and "Anti traits", respectively. Bars represent the value of inhibition percentage against pathogens in dural culture assay. In "Iso Ampli" group, "amplicon" and "isolate" mean 16S rRNA gene sequences obtained via high-throughput sequencing and sanger sequencing, respectively. In "Anti traits" group, "AntiIsolate" and "nonAntiIsolate" mean that isolates used and not used in dural culture assay, respectively. Bars of different colors represent different pathogen strains (*Alternaria alternata* BXL, *A. alternata* DJQ and *Epicoccum nigrum* ACCC37858) used in dural culture assay.

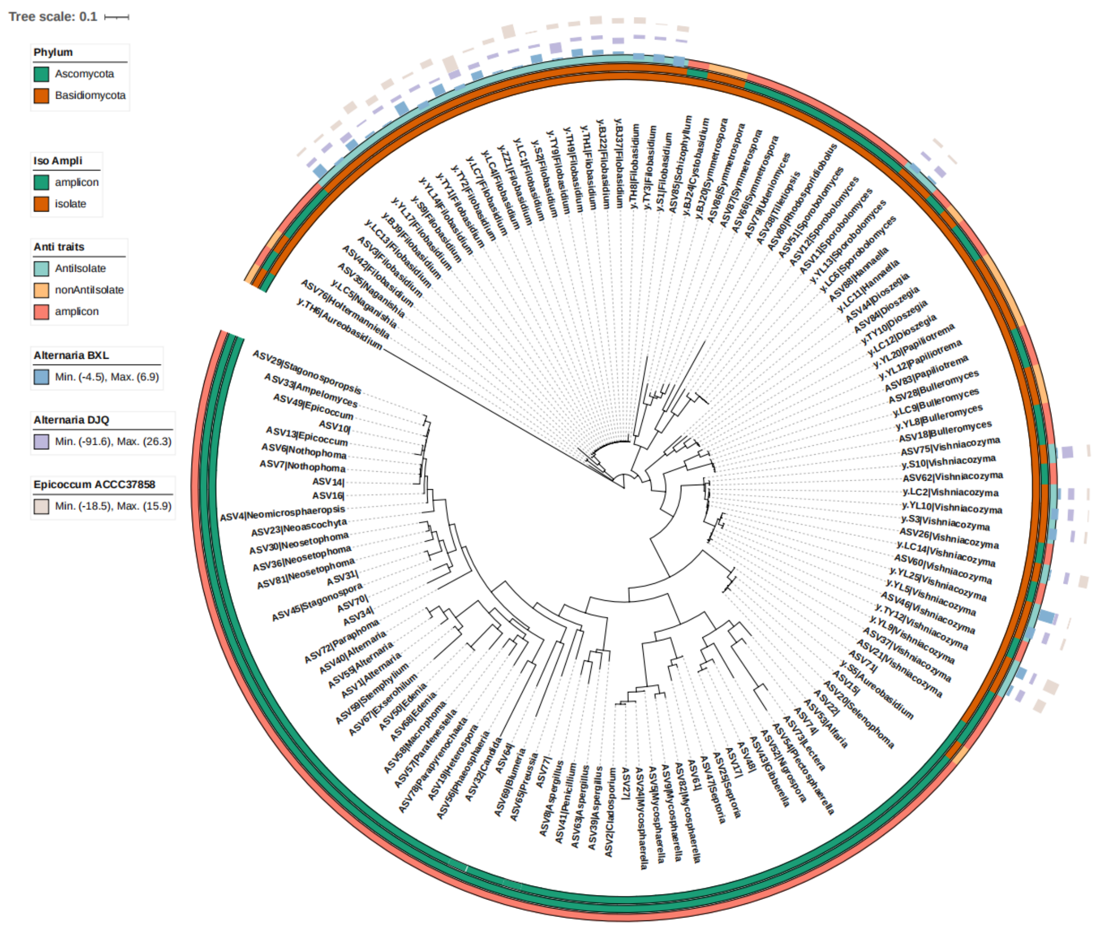

Fig. S10 Phylogenetic tree of 43 yeast isolates and ASVs occurred in more than 50% samples based on ITS sequences. From the inside to the outside, the three rings represent "Phylum", "Iso Ampli", and "Anti traits", respectively. Bars represent the value of inhibition percentage against pathogens in dural culture assay. In "Iso Ampli" group, "amplicon" and "isolate" mean ITS sequences obtained via high-throughput sequencing and sanger sequencing, respectively. In "Anti traits" group, "AntiIsolate" and "nonAntiIsolate" mean that isolates used and not used in dural culture assay, respectively. Bars of different colors represent different pathogen strains (*Alternaria alternata* BXL, *A. alternata* DJQ and *Epicoccum nigrum* ACCC37858) used in dural culture assay.

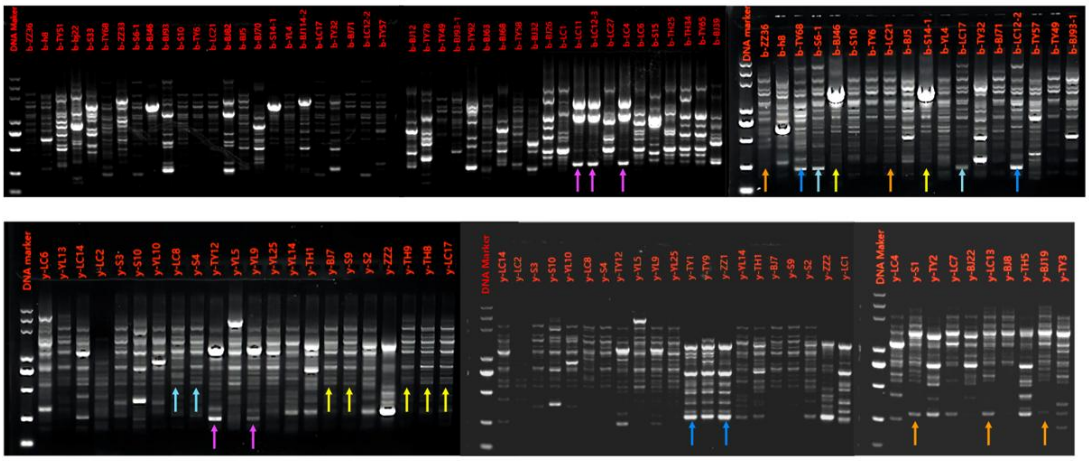

Fig. S11 Fingerprint patterns of the 40 bacterial strains and 32 yeast isolates typed using BOX-PCR. Arrows with the same color represent the same fingerprint pattern of the isolates

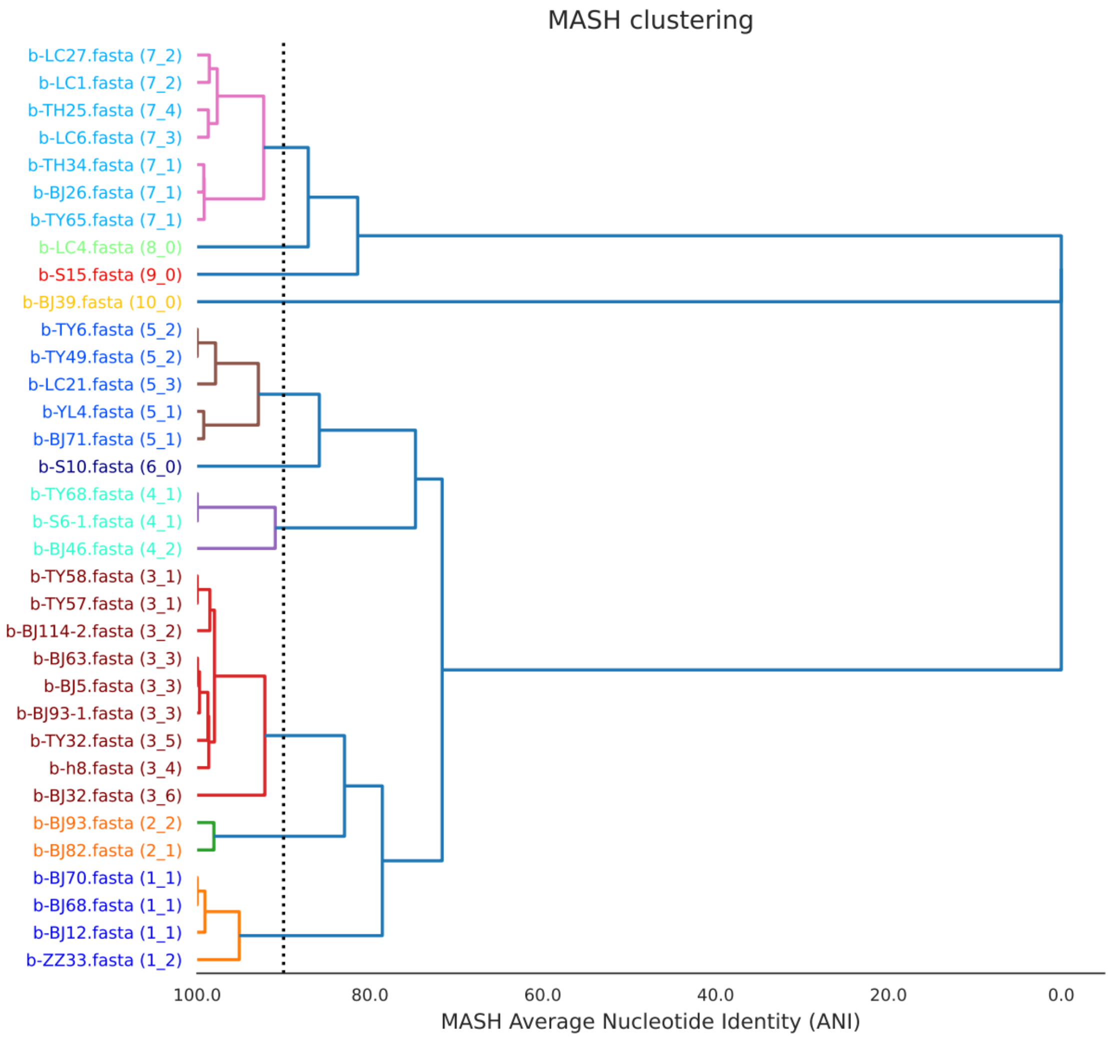

Fig. S12 Average nucleotide identity (ANI)-based phylogenetic tree of 34 bacterial isolates constructed by MASH clustering for the primary clustering dendrogram. The dotted line represents 90% ANI. Different colors represent different primary clusters. The same cluster number in parentheses indicates that the genomes similarity is more than 99% (considered to be the same isolate).
